# Chemical Composition, Antioxidants, Antibacterial, and Insecticidal Activities of *Origanum elongatum* (Bonnet) Emberger & Maire Aerial Part Essential Oil from Morocco

**DOI:** 10.3390/antibiotics12010174

**Published:** 2023-01-14

**Authors:** Imane Tagnaout, Hannou Zerkani, Noureddine Bencheikh, Smail Amalich, Mohamed Bouhrim, Ramzi A. Mothana, Mohammed R. Alhuzani, Rachid Bouharroud, Christophe Hano, Touriya Zair

**Affiliations:** 1 Chemistry of Bioactive Molecules and the Environment, Faculty of Science, University Moulay Ismail, Zitoune Meknes B.P. 11201, Meknes 50050, Morocco; 2 Faculty of Sciences, University Mohammed First, Boulevard Mohamed VI BP 717, Oujda 60000, Morocco; 3 Laboratory of Phytochemistry, National Agency of Medicinal and Aromatic Plants of Taounate, Taounate 34012, Morocco; 4 Laboratory of Biological Engineering, Team of Functional and Pathological Biology, Faculty of Sciences and Technology Beni Mellal, University Sultan Moulay Slimane, Beni-Mellal 23000, Morocco; 5 Department of Pharmacognosy, College of Pharmacy, King Saud University, P.O. Box 2457, Riyadh 11451, Saudi Arabia; 6 Integrated Crop Production Unit, Regional Center for Agronomic Research of Agadir, Agadir 80350, Morocco; 7 Laboratoire de Biologie des Ligneux et des Grandes Cultures, INRAE USC1328, Campus Eure et Loir, Orleans University, 28000 Chartres, France

**Keywords:** *O. elongatum*, EO, GC-MS, antioxidant activity, antibacterial activity, insecticidal activity

## Abstract

The aim of this research is to profile the chemical composition of the essential oil (EO) extracted from the aerial parts of *Origanum elongatum* (*O. elongatum*) and to evaluate its antioxidant, antibacterial and insecticidal activities on *Ceratitis capitata* adults. Gas chromatography coupled with mass spectrometry (GC/MS) revealed a total of 27 constituents in EO of *O. elongatum*, which accounted for 99.08% of its constituents. Carvacrol (57.32%) was a main component, followed by *p*-cymene (14.70%) and *γ*-terpinene (9.84%). The antioxidant activity of *O. elongatum* EO was investigated using DPPH (1,1-diphenyl-2-picrylhydrazyl), FRAP (Ferric reducing antioxidant power), and TCA (the total antioxidant capacity) methods. This EO exhibited a remarkable antiradical and reducing power against DPPH (IC_50_ = 2.855 ± 0.018μL/mL), FRAP (EC_0.5_ = 0.124 ± 0.013µL/mL) and TCA (IC_50_ = 14.099 ± 0.389 mg AAE/g of the EO). The antibacterial tests in vitro, using the disc and dilution methods, were carried out on nine pathogenic bacteria isolated from the hospital patients, such as *Enterococcus faecalis*, *Serratia fonticola*, *Staphylococcus aureus, Acinétobacter baumannii, Klebsiella oxytoca*, *Klebsiella pneumoniae sensible*, *E.coli sensible*, *E.coli resistante*, and *Enterobacter aerogenes*. The EO demonstrated a considerable antibacterial activity with minimum inhibitory concentrations (MIC) from 2 to 8 µL/mL against all strains except *Staphylococcus aureus* (MIC = 32 µL/mL). Regarding the insecticidal activity, the fumigation test indicated a high efficacy (100% mortality), and a lethal dose of LD_50_  =  17 ± 0.53 μL/L air was found after 24 h of exposureTherefore, *O. elongatum* EO could be utilized as a natural antioxidant, antibiotic and biopesticides.

## 1. Introduction

The natural flora is largely made up of medicinal and aromatic plants, which are valued as resources in a number of sectors, including the food, cosmetics, and pharmaceutical industries. The valorization of these natural plant resources essentially involves extracting their essential oils. Generally, they are obtained through steam distillation or hydrodistillation. The oils captured are volatile liquids that containsecondary metabolites characterized by a complex composition and a strong odor. Recent studies have concentrated on the biological characteristics of essential oils, such as their antioxidant, anti-inflammatory, antiviral, and insecticidal effects [1,2,3]. In fact, the many biological properties of essential oils make them very promising preservatives for the food industry [4,5,6]. Utilizing volatile oils in the food industry proves to be a relevant choice for a specific risk of contamination or the need to reduce or replace the agents of chemical or synthetic preservatives.

One of the most significant plant families for the production of essential oils with antioxidant and antimicrobial characteristics is the Lamiaceae family. The majority of aromatic plants that are abundant in essential oils can be found in the Mediterranean region, where the production of these oils is proving to be a lucrative source of ecological and economic development. Among these aromatic and medicinal plants, *Origanum* species are rich in EO and they are consumed worldwide as spices. In addition, they have various biological activities, the potentials of which have been revealed by several scientific studies [7,8,9]. *Origanum* belongs to the Lamiaceae family and it is called “*Oregano*” in English. It is divided into 39 species distributed in the Mediterranean, Euro-Siberian and Irano-Turanian regions [10,11]. Due to its multiple therapeutic and condiment properties, its medicinal use dates back thousands of years. The ancient Greek and Roman empires employed the leaves for the treatment of skin lesions and as an antiseptic, as well as for other ailments such asasthma, diarrhea and indigestion. In Greece, an infusion of oregano is still used as a folk remedy for colds and stomach ailments. In Morocco, the species of oregano have great popularity and are locally known as “*Zaatar*” or “*Zwi*” in Berber. In aqueous infusion, *Zaatar* is traditionally used to treat dysentery, colitis, bronchopulmonary, gastric acidity, and gastrointestinal diseases, while in the Middle Atlas (Jbel-Bouiblane), this species is used for the treatment of liver disorders [12].

We were interested in the phytochemical and biological studies of the species called *Origanum elongatum* (Bonnet) Emb. & Maire. It is an endemic perennial plant that grows in the mountains of the Rif and Middle Atlas. It grows in shale soils and at an altitude of between 400 and 1500 m above sea level. The lightness of the inflorescences, their abundance, and the staggered nature of the flowering can providean ornamental interest to this species. In the past, its essential oil was artisanally distilled and sold commercially under the name of Rif thyme essence. In Moroccan traditional medicine, *O. elongatum* is used as an infusion to treat hepatic diseases, and it is very much visited by the bees. The aim of the current investigation was to determine the chemical content of the essential oil of *O. elongatum* and to study its antibacterial and antioxidant activities. Therefore, these findings can be applied to additional applications of the EO of *O. elongatum* as a valuable natural product in the pharmaceutical and food industries.

## 2. Results and Discussion

### 2.1. The Yield of the Essential Oil

Based on the dry plant matter of the plant’s aerial portion, the average yield of the essential oil was estimated. Indeed, the aerial part of *O. elongatum* appears clearly rich in EO with a yield of 4.46 ± 0.1%. This result remains superior to those reported by several researchers [13,14,15]. By comparing these results with other species, our samples of *Origanum elongatum* presented the highest yield of the essential oil compared to *Origanum compactum* (2.10%), and *Origanum vulgare* (2.5%) [16]. El Harsal et al. (2018) have investigated the influence of the extraction time on the yield of the EO of *O. elongatum* using hydrodistillation as an extraction process [17]. They argue that 140 min is the optimal duration to achieve a maximum yield of the EO for the studied species. *O. elongatum* economic exploitation is justified by the relatively high essential oil output it produces when compared to other plants.

### 2.2. Chemical Composition of O. elongatum Essential Oil

**Figure 1:**
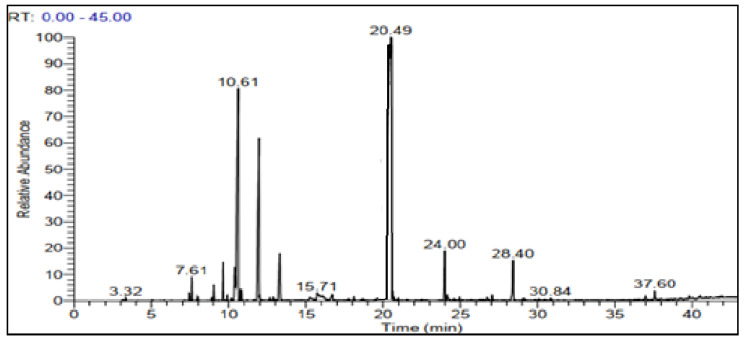
Chromatogram of the *O. elongatum* EO.

**Figure 2:**
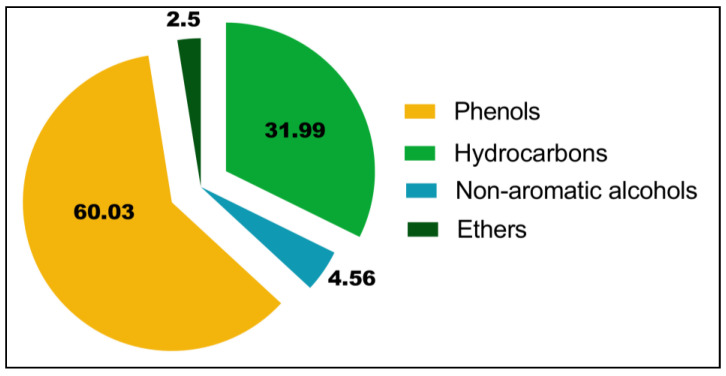
Percentage of the main chemical families of the EO of *O. elongatum*.

The chemical analysis of the EO of *O. elongatum* using gas chromatography coupled with mass spectrometry presented in Figure 1 enabled the detection of 27 chemical compounds, accounting for 99.08% of the overall chemical composition (Table 1). The chemical profile of the EO showed the dominance of oxygenated monoterpenes (65.08%) and hydrocarbon monoterpenes (29.06%), while the hydrocarbon and oxygenated sesquiterpenes were found in low percentages (2.93% and 2.01%, respectively). This essential oil was mostly composed of carvacrol (57.32%), followed by two monoterpene hydrocarbons with much lower contents: p-cymene (14.7%) and *γ*-terpinene (9.84%). The other identified constituents were present with contents comprised between 2.94 and 0.75%. This is a decreasing order of abundance of linalool (2.94%), thymol (2.71%), (Z)-caryophyllene (2.38%), caryophyllene oxide (2.01%), myrcene (1.43%) and *α*-pinene (0.75%). In addition, we remark the presence of thymohydroquinone in very low quantities (0.29%). Based on the results shown in Figure 2, we can observe that the EO of *O. elongatum* is rich in phenols (60.03%) and hydrocarbons (29.06%). In addition, we note the presence, in lower proportion, of non-aromatic alcohols (4.56%) and ethers (2.5%). However, the rate of the compounds identified in the analyzed EO varies significantly with the literature. These differences in chemical composition are dedicated to several factors, such as origin, growth stage, environmental influences and genetic heritage. In general, the impact of these factors on biosynthetic pathways causes variations in the majority of distinctive chemicals’ qualitative and quantitative properties, which results in the existence of distinct chemotypes that identify EO from various sources. The identified chemical composition presents similarities with several previous studies established in the north of Morocco. A review of the phytochemistry of *O. elongatum* extracts indicates that the EOs of different parts of this plant are rich in terpenoids such as *α*-tujene, *β*-myrcene, *p*-cymene, *γ*-terpinene, linalool, terpinene-4-ol, thymol, carvacrol, *β*-caryophyllene, *β*-bisabolene, caryophyllene oxide, *α*-terpinene, limonene, thymoquinone, and thymohydroquinone, *α*-phellandrene, caryophyllene, 3-carene, and *α*-pinene [18]. According to the same authors, thymol, carvacrol, *p*-cymene, and *γ*-terpinene are compounds found in the EOs of all parts of *O. elongatum*, which consists of the chemical composition of our oil from which we have detected these terpenoids as the major composition of the oil in question. Indeed, Cosentino et al. (199) found that this essential oil is mainly composed of carvacrol (62.8–79.2%), *γ*-terpinene (0.6–7.3%) and *p*-cymene (5.2–16.9%) [19]. However, Bellakhdar et Il Idrissi (1990) reported that the EO of *O. elongatum* from the Middle Atlas are rich in thymol (60%) [20].Figuérédo et al. (2006) have reported that the essential oil of the species harvested from the Rif region is rich in carvacrol (79.2%) and the essential oils of the cultivated plants of *O. elongatum* are similarly dominated by carvacrol (56, 1–63.9%) [21]. El Moussaoui et al. (2013) determined the composition of the essential oils isolated from the leaves and flowers of *O. elongatum* harvested from the north of Morocco [13]. This comparative study showed that the essential oils of the leaves and flowers of *O. elongatum* contain the same compounds but with variable proportions. Indeed, the four main compounds identified are carvacrol (19.21% in the leaves and 40.12% in the flowers), thymol (3.57–14.24%), *p*-cymene (16.08–16.19%), and *γ*-terpinene (7.27–13.48%). Using hydrodistillation as an extraction process, El Harsal et al. (2018) have demonstrated the influence of the extraction time on the chemical composition of the EO isolated from *O. elongatum* harvested in the north of Morocco [17]. The chemical profile of the essential oil exhibits the predominance of the oxygenated compounds (65.14%) and hydrocarbon compounds (28.02%). Overall, the main constituents of the EO werethymol (63.44%), *γ*-terpinene (14.63%), *p*-cymene (9.56%), *β*-caryophyllene (2.33%) and *α*-terpinene (1.65%). Moreover, the chromatographic and spectrophotometric analyses performed by Ramzi et al. (2017) on the essential oils of *O. elongatum* of five populations originating from the Rif mountains, harvested in different vegetative stages, reported the presence of the same chemical composition in the EO with modest variations between the flowering and fruiting phases and origin [14]. The majority compounds are carvacrol (67.34–81.72%), *γ*-terpinene (3.29–10.75%), *p*-cymene (3.62–7.81%) and thymol (1.79–9.17%). Bakhaet al. (2018) have established a study of the intraspecific chemical variability of the EO from *O. elongatum* harvested in the Rif and Middle Atlas mountains [15]. They confirm the dominance of oxygenated monoterpenes such as carvacrol (10.52–77.45%), thymol (0.98–62.40%) and *p*-cymene (7.63–31.53%). Thus, elevated rates of *γ*-terpinene (1.72–4.98%) and linalool (1.51–2.88%) have been reported in some samples. They suggest the existence of four chemotypes: carvacrol, carvacrol/thymol, carvacrol/*p*-cymene and thymol as the main components. It is seen that the carvacrol chemotype was the most distributed in the studied geographical area, and it is the same chemotype encountered in our sample. An analogous composition was reported by Oualili et al.(2020)for the EO of *O. elongatum* harvested from Targuist in the Rif of Morocco, who noted that the major compounds are carvacrol (60.42%), *p*-cymene (13.9%), and *γ*-terpinene (9.4%) [22]. Using the gas chromatography coupled with mass spectrometry, Oumam et al. (2021) identified sabinene (43.92%), *γ*-terpinene (16.93%), carvacrol (9.71%), isoterpinolene (8.74%) and thymol (5.53%) as the major componentsof the EO of the *O. elongatum* leaves of the northern region of Morocco [23]. In overall, the chemical composition of EOs from different parts of *O. elongatum* remains nearly identical, albeit at varying concentrations. This variability could be attributed to the geographical origin of the samples, climatic and ecological conditions, harvest season and drying process, and extraction procedures [24,25,26].

### 2.3. Antioxidant Activity of the Essential Oil of O. elongatum

**Figure 3:**
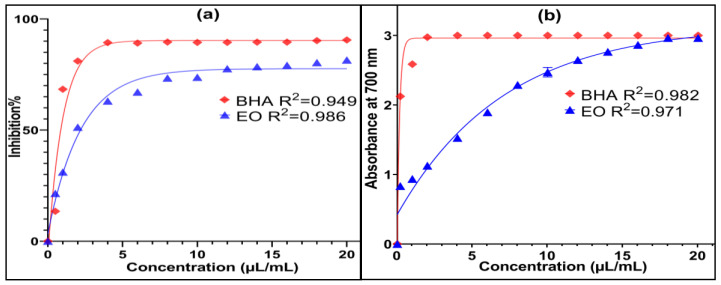
Antioxidant activity of the EO of *O. elongatum* and BHA standard by DPPH (**a**) and FRAP (**b**) methods.

**Figure 4:**
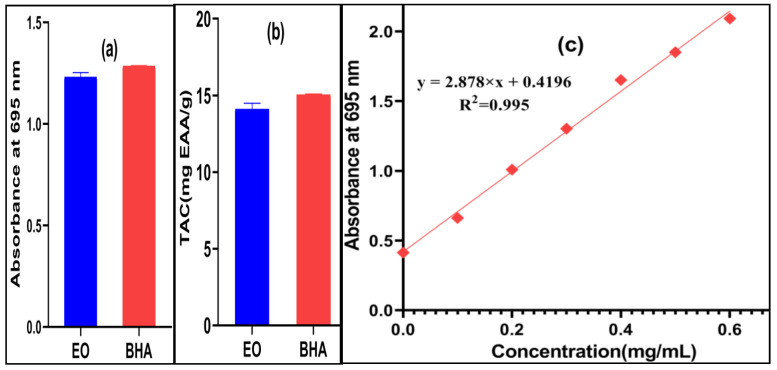
(**a**)Absorbance at 695 nm, (**b**) total antioxidant capacity of the EO of *O. elongatum* and BHAstandard; (**c**) Regression curve of ascorbic acid.

The results of the antioxidant activity of the EO of *O. elongatum* of Middle Atlas (Bouyblane) and standard (BHA) evaluated using three methods, DPPH, FRAP and TCA, are presented in Figure 3 and Figure 4.The antioxidant power of the EOs has been widely studied. It has been valued as a potential natural substitute for the synthetic antioxidants used in specific sectors for the preservation of foods stuffs. For comparative reasons, the values of the IC_50_ and EC_0.5_ of the antioxidant activity of the EO of *O. elongatum* and BHA standard were determined from the graphs for the DPPH and FRAP tests (Table 2). In the first method (Figure 3a), the EO of the studied oregano and BHA were able to reduce the stable DPPH radical which results in the color change from purple to yellow. The EO of *O. elongatum* shows a lower activity than standard BHA, with IC_50_ values of 2.855 ± 0.018µL/mL and 1.512 ± 0.005 mg/mL, respectively. Oualili and others (2020) have revealed a strong antioxidant activity of the EO of *O. elongatum* from the Rif, noting IC_50,_ which equals 1.20 g of extract/g of DPPH. Indeed, they showed that the carvacrol is directly involved in this activity [22]. Moreover, El Babili et al. (2011) found that the essential oil of *O. compactum* from Morocco has a significant antioxidant power with an IC_50_ of 60 mg/L [16]. For the FRAP method, the presence of reducers in the medium results in the reduction of the complex Fe^+3^/ferric cyanide of the yellow color to the ferrous form of greenish blue color through the donation of an electron. The increase in the absorbance at 700 nm indicates an increase of the reduction capacity. According to the FRAP tests, we find that the EO of *O. elongatum* has a slightly higher antioxidant power (EC_0.5_ = 0.124 ± 0.013µL/mL) than that of the standard BHA (EC_0.5_ = 0.15 ± 0.002 mg/mL). The results obtained using the FRAP method (Figure 3b) demonstrate that the EO of *O. elongatum* is able to reduce the ferric ions (Fe^3+^) easily and therefore neutralize the free radicals by donating electrons. Concerning the third method, phosphomolybdenum is quantitative since the total antioxidant activity is expressed as the number of equivalents of ascorbic acid. The optical density and the antioxidant capacity of the essential oil of *origanum elongatum* are presented in Figure 4. *O. elongatum* oil had TAC of 14.099 ± 0.389 mg AAE/g of EO. As compared to the synthetic antioxidant BHA (15.041 ± 0.05 mg AAE/g), *O. elongatum* oil exhibited approximate total antioxidant capacity. The results suggest that *O. elongatum* oil could be used as a natural antioxidant in the food systems. Given that the unfavorable side effects of synthetic antioxidants, such as liver damage or carcinogenesis, are well-known, these results appear encouraging for the preservation of food [27].Alcohols, phenols, terpenes, and ketone molecules, which serve as antagonistic or synergistic components of essential oils, combine chemically to produce antioxidant activity. In fact, numerous studies have linked the phenol content to the plant’s essential oil’s antioxidant power [28,29]. The great antioxidant potential of the EO extracted from *O. elongatum* is linked to the dominance of the oxygenated and hydrocarbon monoterpenes, the majority compounds of which are carvacrol (57.32%), *p*-cymene (14.70%) and *γ*-terpinene (9.84%), without neglecting the minority presence of certain families, such as non-aromatic alcohols and ethers, or the synergy between them. Several studies have attributed the antioxidant potential of the oregano species to the high content of phenolic compounds such as the carvacrol and thymol as they are able to scavenge free radicals through their phenolic hydroxyl groups [30,31]. The carvacrol has several biological and pharmacological activities: antioxidant, antibacterial, antifungal, anticancer, anti-inflammatory, hepatoprotective, spasmolytic and vasorelaxan [8,32,33]. The evaluation of the antioxidant capacity of the thymol, carvacrol and *γ*-terpinene compared to that of the synthetic antioxidant Trolox showed that carvacrol and thymol have an antioxidant activity similar to that of Trolox [34]. Furthermore, the antioxidant capacity of carvacrol was significantly higher than the same concentration of its thymol isomer [34,35]. In addition, the essential oils that are composed of oxygenated monoterpenes and/or sesquiterpenes are recognized by strong antioxidant properties [36].

### 2.4. Antibacterial Activity of the EO of O. elongatum

Three categories can be used to describe the antibacterial activity of the EO:(i) weak activity (inhibition zone ≤12 mm), (ii) moderate activity (12 mm < inhibition zone <20 mm) and (iii) strong activity (inhibition zone ≥ 20 mm). The results of the antibacterial activity of the essential oil of *O.elongatum* are recorded in Table 3. The statistical analysis showed that the diameters of inhibition were significantly different for the EO (*p* < 0.05). The tests of the diffusion performed with a volume of 5 µL showed that the tested EO exerts an antibacterial activity against the most tested bacteria with inhibition zone diameters ranging from 9 ± 0.00 to 48.05 ± 0.95 mm. The EO isolated from the aerial part of the oregano exhibited a great antibacterial activity against all tested strains, especially against sensitive *K.oxytoca, S. fonticola*, *E. coli, E. aerogenes, E. faecalis, A. baumannii*, showing zones of inhibition that varied from 20.7 ± 1.20 mm to 48.05 ± 0.95 mm. On the other hand, a moderate activity was observed against sensitive *K. pneumoniae* and resistant *E. coli* with zones of inhibition ranging from 14.15 ± 0.15 mm to 19.95 ± 0.07 mm. *Staphylococcus aureus* was an exception, having revealed resistant activity to oregano oil. Compared with the antibiotics timentin TIM85, cefoxitin FOX30 and piperacillin PRL100 used as controls, the EO of oregano showed a more pronounced inhibitory action. The strains that have shown a resistance to the antibiotic action are vulnerable to the action of the EO of the studied oregano; this is the case for the bacteria: *E. faecalis, S.fonticola, K. oxytoca,* sensible *E.coli* and *E. aerogenes*. Our results agree with those of Moussaoui et al.’s (2013) study, where they have reported that the essential oils extracted from the flowering tops and leaves of *O. elongatum* (diameters of inhibition 18.00 ± 0.00 mm) exhibited a moderate antibacterial activity against *E. coli* O157:H7 (diameters of inhibition 19.67 ± 1.15 mm) [13]. Our findingsare further confirmed by the study of El Harsal et al. (2018) [17]. On the one hand, the EO of *O. elongatum* expressed a moderate antibacterial power against *E.coli* K12 (16.00 ± 1.00 mm). On the other hand, it exerted great activity against *S. aureus* ATCC 25923 and *E. coli* ATCC 25922 with diameters of inhibition of 27.00 ± 1.73 and 21.33 ± 0.57 mm, respectively. In addition, the existence of a linear correlation between the inhibition zones and the content of thymol, which is the main compound in each fraction of EO of *O. elongatum*, was found. Numerous studies investigated the antibacterial effect of the EO of different species of the genus *Origanum*. Bouyahya et al. (2017) reported the antibacterial activity of the EO extracted from the aerial part of *O. compactum* collected from the north of Morocco in three phenological stages: vegetative, flowering and post-flowering [37]. The EO extracted at the flowering stage with the carvacrol (43.584%) and thymol (10.33%) as major compounds revealed a remarkable antibacterial activity against *S. aureus* MBLA (35 ± 0.94 mm), *E. coli* K12 (31 ± 0.91 mm) and *L. monocytogenes* (21 ± 0.33 mm). Bouhdid et al. (2008) have studied the EO of *O.compactum*; the authors have found a powerful inhibitory activity against *Staphylococcus aureus* (27 mm), *Bacillus subtilis* (25 mm), *Proteus mirabilis* (32 mm), *Escherichia coli* K12 (20 mm) and *Escherichia coli* serovarO157:H7 (20 mm) [28]. Moreover, the EO extracted from the aerial part of *O. glandulosum* in Algeria showed significant antibacterial power against *K. pneumoneae* (16.4 mm), E. coli (22.6 mm) and *S.aureus* (25.6 mm) [38].

The determination of MIC and MBC performed for the EO of *O. elongatum* demonstrated a well-defined promising activity. The results of the test are given in Table 2. From the results, we can see that the values of MIC confirm the results of the diffusion method. In these tests, the EO of *O. elongatum* recorded the lowest MIC of 2 µL/mL against sensitive *K.pneumoniae*(Table 4). In contrast, the largest MIC of 32µL/mL is reported by this EO against resistant *S. aureus* (Table 4). The strains *E. faecalis, E. aerogenes, S. fonticola,* and *A. baumannii* were inhibited at the same MIC, which are equal to 4 μL/mL.On the other hand, for the resistant *K. oxytoca* and *E. coli* strains, the EO of the studied oregano is only active from 8 μL/mL and 16 μL/mL, respectively. Concerning MBC, the minimum concentration of 4 μL/mL was obtained by this EO against the strains: sensitive *A. baumannii, E. coli* and sensitive *K. pneumoniae.* The highest concentration of 32 μL/mL was remarked against the resistant strains *S.aureus* and *E. coli*. The EO of the studied oregano induces a strong decrease in the bacterial growth and leads to the cell death of the strains *E.aerogenes* and *S. fonticola* at the same concentration of 8µL/mL. For *K. oxytoca* strain, the EO has a bactericidal character at a concentration of 16 μL/mL. The MBC/MIC ratio allows the defining of the bacteriostatic or bactericidal character of an essential oil. When this ratio is less than 4, the oil is considered bactericidal. The EO of the studied oregano exerts a bactericidal effect against the following bacteria: *E.aerogenes*, sensitive *K.pneumoniae, E. faecalis,* resistante *S.aureus, S. fonticola, K. oxytoca* and *A. baumannii,* resistant and wild-type *E. coli*. These results highlighted the bactericidal power of the EO of *O. elongatum* against the studied bacteria. Similar results have been previously reported for the obtained EO of oregano from different species [13,28,29,38,39,40,41]. Furthermore, Oumam et al. (2021) demonstrate the in vitro antibacterial activity of *O. elongatum* essential oil from the Northern Moroccan region [23]. These authors indicate that the oil of this plant has antibacterial activity against *S. aureus* (ATCC 6538209P), *S. aureus* meticillin-resistant (T32370), *K. pneumoniae* (A9d), *S. Aboni* (CIP 8039), *E. coli* (CIP 53126), and *P. aeruginosa* (CIP 76110), with MIC values ranging from 200 to 3200 µg/mL, which is consistent with our results. In addition, El Moussaoui et al. (2013) reported that the EO isolated from the leaves and flowering tops of *O. elongatum* collected from the north of Morocco are dominated by the presence of two phenolic compounds: carvacrol (19.21–68.63%) and thymol (14.2–43.57%) [13]. These EOs presented a MIC and MBC varying between 0.125 and 0.5% against *E. coli* O157. El Harsal et al. (2018)have noticed that the fractions of the EO of *O. elongatum* extracted between 80 and 160 min have greater antibacterial activity than the fractions recovered at the start of the distillation and the total EO. The fractions of the EO recovered between 141 and 160 min mainly composed of the thymol (86.09%) have the strongest antibacterial activity with, values of MIC comprised between 0.0312 and 0.125% (*v/v*), and MBC of 0.0312 at 0.25% (*v/v*) against *E. coli* K12 MBLA, *E. coli* ATCC 25922 and *S. aureus* ATCC 25923. In the study of Bouyahya et al. (2017), the antibacterial activity of the EO extracted fromleaves of *O. compactum,* harvested at the flowering stage, mainly composed of carvacrol (43.584%) and thymol 10.33%, revealed MIC and MBC of 0.0312% against *S. aureus* MBLA and 0.0625% for *E. coli* K12 [42].The authors of this latest study linked the observed inhibitory effect for the main compounds to the synergistic effects between these components and to the additive effects of the minor compounds, which can enhance the antibacterial action. Another study testifies to the significant antibacterial effect of the EO of *O. compactum* from Morocco against *Escherichia coli*, *Bacillus subtilis*, *Staphylococcus aureus* and *Listeria innocua*, inducing diameters of inhibition zones varying from 10.33 to 49.00 mm, while the values of MIC and MBC vary from 0.06 to 0.25% (*v/v*) and from 0.12 to 0.5% (*v/v*), respectively [43]. In the same context, Ouedrhiri et al. (2016)demonstrated that the essential oils extracted from *compactum* and *Origanum majorana* have an antibacterial effect against *Staphylococcus aureus* ATCC 29213, *Escherichia coli* ATCC 25922, *Bacillus subtilis* ATCC 3366 and *P. aeruginosa* ATCC 27853, examined using the disk diffusion and microdilution method [44]. In our investigation, the inhibitory activity of the EO of the studied oregano is mainly due to the phenolic action of the carvacrol and thymol. According to [19], when the rates of the phenol are high, the antibacterial effectiveness of the essential oils isgreater. In fact, several authors have pointed out the antimicrobial activity of carvacrol and thymol against *E. coli* in vitro experiences [27,45]. Ultee et al.(1999) consider carvacrol to be a biocide that causes a disturbances in the bacterial membrane leading to intracellular leakage of ATP ions, potassium ions and ultimately cell death [46]. The possible synergistic and/or antagonistic effects of the oil’s minority components should also be considered, as the predominance of monoterpene constituents does not generally signal the highest antibacterial activity for the majority of the tested strains.

### 2.5. Insecticidal Activity

**Figure 5:**
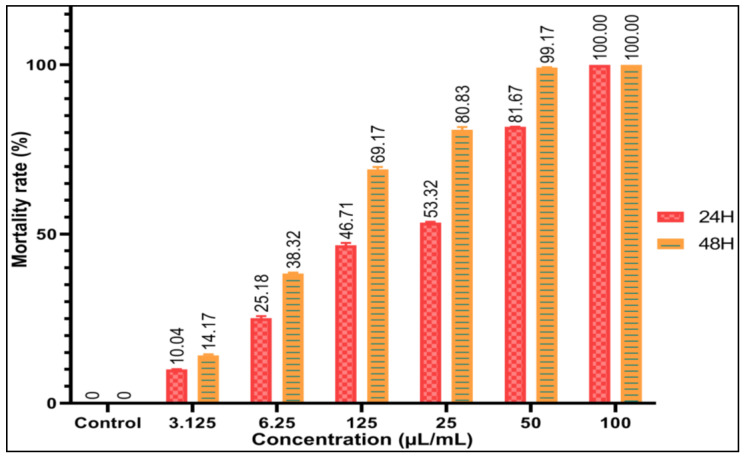
Mortality rate of adults of *C. capitata* at different concentrations of EO of *O. elongatum* after 24 and 48 h of exposure (The results are significantly different (*p* < 0.0001).

Insecticidal activity of *O. elongatum* EO was testedthrough fumigation. The EO tested at increasing concentrations presented a significant toxicity towards the adults of *C.capitata*. Figure 5 illustrates the percentages of mortality of the adults of *C. capitata* after application of the *Oregano* EO at different doses and after exposure times of 24 h and of 48 h. The results showed that the mortality rates (24 h and 48 h) of the control are almost non-existent for the adults of *C. capitata*. In fact, the survival rate of treated flies falls as the EO concentration increases. After 24 h of treatment, the mortality rate of adults of *C. capitata* was 100% from the lethal concentration of 100 µL/mL of *O. elongatum* EO. However, the lowest concentration induced only 10.04% mortality. For 48 h exposure, EO of oregano showed 99.17% mortality at a concentration of 50 μL/mL (Figure 5), while the concentration of oregano EO at 25µL/mL caused mortality rates of 80.83% and 53.32%.The values of LD_50_ of the EO of *O. elongatum* are shown in Table 5. We noticed that the values of LD_50_ of the EO tested vary in function of the duration of exposure. After 24 h and 48 h of exposure, the tests carried out on the adults of *C. capitata* showed that the LD_50_ of the EO of aerial parts of *O. elongatum* were 17 ± 0.53 and 10 ± 0.23µL/mL, respectively. The insecticidal effect of the EO of *O. elongatum* is most likely due to its chemical composition, which includes carvacrol as a major compound. This EO, as was shown, is rich in *monoterpenoids*, which consider insecticidal against various species of insects [47]. Moreover, Lima et al. (2011) reported that the toxicity of EOs on insects is induced by the action of their main compounds [48]. On the other hand, several authors such as Yakhlef et al. (2020), and Benchouikh et al. (2015) indicated that the insecticidal effect of EO is not limited only to the majority compounds, but it could be due to the synergistic action of several minority compounds [49,50]. According to the chemical composition of EO of *O. elongatum*, we remark its abundance in major compounds known for their insecticidal activities, as is the case for carvacrol, thymol, *p*-cymene, *γ*-terpinene. These compounds have been described to be toxic for several species of insects through various studies such as [51,52,53,54,55]. In a previous study, Ramzi et al. (2017) have found that the EOs of *O. elongatum* are rich in carvacrol(67.34–81.72%)and *γ*-terpinène (3.29–10.75%), which have toxic effects on *Varroa destructor* Anderson &Trueman [14]. In this study, the EOs which had percentages of carvacrol around 80, 58 and 69, 40% indicated efficacy rates of 81.80 and 77.27%, respectively against *Varroa mites*. In addition, these researchers revealed the synergistic effect between the two EOs extracted from *T. satureioides and O. elongatum*, which contain high percentages of carvacrol (55.35%) and borneol (20.60%) against the same mite. This mixture was considered the most effective treatment among all the others, as after one day of exposure, it caused a high mortality rate of 93.94%. This study corroborates the insecticidal activity of our EO of *O. elongatum*. However, factors that influence the severity of the action include its chemical makeup, insect type, and length of exposure.

## 3. Materials and Methods

### 3.1. Plant Material

The aerial parts of *O. elongatum* (stems, leaves, and flowers) were sampled in August 2018 while in full bloom on Bouyblane mountain in the Middle Atlas, Morocco. This species identification was performed at the laboratory of botany and plant ecology of the Scientific Institute of Rabat. After identification, a specimen of the plant was deposited in the herbarium of Mohammed First University, Oujda, Morocco, under voucher number HUMPOM517. The various plant parts were dried in the shade for thirteen days.

### 3.2. Extraction of the Essential Oils

The volatile oil of *O. elongatum* was extracted through a hydrodistillation technique utilizing a Clevenger-type device for three hours [56]. The process is repeated three times for each sample of 100 g of the plant material. The obtained essential oils are dried on sulfate of anhydrous sodium, and then stored at a temperature of 4 °C in the dark until their use.

### 3.3. Gas Chromatography Coupled with Mass Spectrometry Analysis of Essential Oils

A gas chromatograph of the Thermo Electron type (Trace GC Ultra) and a mass spectrometer system of the Thermo Electron Trace MS type were used for the chromatographic examination of EOs (Thermo Electron: Trace Ultra GC, Polaris Q MS). The fragmentation was achieved with an electronic impact intensity of 70 eV. A DB-5 column (5% phenyl-methyl-siloxane) (30 m × 0.25 mm × 0.25 m film thickness) and a flame ionization detector (FID) powered by a mixture of He gas/air were installed in the chromatograph. For 5 min, the column temperature was designed to rise at a rate of 4 °C/min from 50 to 200 °C. The used carrier gas was nitrogen, flowing at a rate of 1 mL/min in the split injection mode (leak rate: 1/70). By comparing the essential oil compounds’ Kováts index (KI) and Adams to those of the reference products noted in the literature, the essential oil compounds were identified [57,58]. Additionally, the mass spectra and indexes of each of these compounds were compared to those in the aforementioned databases [59]. The retention duration of any product is compared using the Kováts index to a linear alkane with the same carbon number.

### 3.4. Determination of the Antioxidant Activity of the Essential Oil of O. elongatum

#### 3.4.1. Antiradical of DPPH Method

The antiradical activity of the essential oil of *O. elongatum* was studied using the method based on DPPH (1,1-diphenyl-2-picrylhydrazyl) as a relatively stable radical. The solution of DPPH was prepared by dissolving 2.4 mg of DPPH in 100 mL of ethanol. Different concentrations of the EO (1–20 µL/mL) extracted from *O. elongatum* were prepared by dissolution in absolute ethanol. The test was performed by mixing 200 µL of each concentration with 2.8 mL of the solution of DPPH. These same concentrations were prepared with butylhydroxyanisole (BHA) to serve as positive controls. In addition, a blank was performed with absolute ethanol alone. Then, the samples were left in the dark for 30 min, and the discoloration compared to the negative control containing only the solution of DPPH was measured at 517 nm. The results were expressed as a reduction percentage of DPPH (AA%):

$$\mathrm{AA}\%\;=\frac{A_{\mathrm{control}}-A_{\mathrm{sample}}}{A_{\mathrm{control}}}\times 100$$

AA%: percentage of the antioxidant activity, Acontrol: absorbance of the solution containing only the solution of DPPH• radical, and Asample: absorbance of the solution of the samples to be tested in the presence of DPPH.

The graph presents the variation of the absorbance as a function of the concentration of the extract permitted to determine IC50. The values of the concentrations to inhibit or reduce 50% of the initial concentration of DPPH (IC50) were determined graphically using a linear regression. Since a compound’s antioxidant capacity cannot be measured in absolute terms, the results are frequently compared to an antioxidant reference, such as butylated hydroxytoluene (BHT) or butylated hydroxyanisole (BHA).

#### 3.4.2. Method of the Reduction of Iron: FRAP (Ferric Reducing Antioxidant Power)

The method proposed by [60], is used to test the ability of phenolic extracts to convert ferric iron (Fe^3+^) present in the potassium ferricyanide complex to ferrous iron (Fe^2+^). In a test tubes, 1 mL of the essential oil of *O. elongatum* diluted in absolute ethanol at different concentrations (1–20 µL/mL) is mixed with 2.5 mL of a solution of the phosphate buffer at 0.2 M (pH 6.6) and 2.5 ml of a solution of potassium ferricyanide K_3_Fe (CN)_6_ at 1%. The set is incubated in a water bath at 50 °C for 20 min. Then, 2.5 mL of trichloroacetic acid at 10% is added to stop the reaction. For 10 min, the set is centrifuged at 3000 rpm. After that, 2.5 mL of the distilled water, 2.5 mL of each concentration’s supernatant, and 0.5 mL of the FeCl3 aqueous solution containing 0.1% are combined. To calibrate the UV–VIS spectrophotometer, the reaction medium’s absorbance is measured at 700 nm in comparison to a similarly prepared blank, but with distilled water in place of the essential oil. The standard antioxidant solution butylated hydroxyanisole (BHA), whose absorbance was measured under the same circumstances as the samples, serves as the positive control. The increase in absorption corresponds to an increase in the reducing ability of the tested essential oils.

#### 3.4.3. Total Antioxidant Activity by Phosphomolybdenum Method

According to the method described by [61], the total antioxidant capacity (TAC) of the essential oil was evaluated. One milliliter of the reagent solution was added to an aliquot of 100 L of each essential oil that had been diluted in ethanol (0.6 M sulfuric acid, 28 mM sodium phosphate, and 4 mM ammonium molybdate). For 90 min, the tubes were incubated at 95 °C in a bath of boiling water. The absorbance of the aqueous solution of each sample was measured in the spectrophotometer at 695 nm after the samples were cooled to room temperature (Genesys 10 UV, Thermo Scientific, Bordeaux, France). BHA was employed as the BHA standard, and the results were represented as equivalents of ascorbic acid (mg/g) of the oil.

### 3.5. Determination of the Antibacterial Activity

#### 3.5.1. Bacterial Strains

We have chosen nine strains of the bacteria, which are frequent in human pathology belonging to the category of the gram-negative. These bacterial strains are: *Escherichia coli* sensitive, *Escherichia coli* resistant, *Enterobacter aerogenes*, sensitive *Klebsiella pneumonia*, *Enterococcus faecalis*, *Serratia fonticola*, *Staphylococcus aureus*, *Acinétobacter baumannii*, and *Klebsiella oxytoca*. They were provided by the laboratory of bacteriology of the Mohammed V hospital center of Meknes, Morocco, isolated from the patients, and then preserved in a subculture on a specific agar media. These bacterial species frequently cause nosocomial infections, which represent a serious public health issue. One of the key reasons why treatments do not work for these bacteria is the development of multi-resistance.

#### 3.5.2. Agar Disk Diffusion Assay

Disk diffusion was used to conduct the test in an agar medium (Mueller Hinton Agar). In short, the microorganisms that have grown on nutrient agar and been incubated at 37 °C for 18 h were suspended in a saline solution (NaCl) at 0.9% and adjusted to a turbidity of 0.5 Mac Farland standards (108 CFU/mL). The suspension was inoculated into Petri dishes of a diameter of 90 mm using a sterile non-toxic cotton swab on a wooden applicator. The dissolution of the essential oils was performed in 0.5% (v/v) of DMSO. According to our experiences, the latter does not affect the growth of the bacteria. The discs of the sterile paper of a diameter of 6 mm impregnated with 5 μL of the studied EO were placed in the Petri dishes and incubated during 24 h at T = 37 °C. The sensitivity of the investigated strains was assessed using the commercial antibiotics as a positive reference. Finally, the diameters of zones of inhibition (DZI)) were determined to ascertain the antibacterial characteristics of the explored EO.

#### 3.5.3. Determination of the Minimum Inhibitory Concentration (MIC) and the Minimum Bactericidal Concentration (MBC) in the Liquid Medium

A bacterial culture that has been incubated for 18 to 24 h was used to create a suspension of bacteria with a density that was equal to the 0.5 Mac Farland standard (108 CFU/mL). In order to create a stock solution, the essential oil and dimethyl sulfoxide were combined (*v/v*) (DMSO is the chosen emulsifier). The dilution of the used essential oil was 70% (70% of the EO, 30% of DMSO). A test tube containing 4 mL of the liquid culture medium was filled with a volume of 40 µL of the bacterial suspension. Then, we aseptically added various volumes of the emulated essential oil to achieve a rise in the essential oil final concentrations of the from C1 to C9 (1, 4, 8, 16, 32, 64, 128, 256 and 512 µL/mL). A tube of a negative control was prepared and contained only the suspension and culture medium. Three repetitions were made. The set was vortexed for homogenization, and then incubated in an oven at a temperature of 37 °C for 24 h. After 24 h of incubation at T = 37 °C, the lowest concentration of the essential oil inhibited any increase visible to the naked eye, which was noted as the minimum inhibitory concentration (MIC). Furthermore, the MBC was assessed by inoculating 100 μL of each tube, the concentration of which is greater than or equal to the MIC on a solid medium (MHA). The concentration of the lowest essential oil at which 99.99% of the bacteria are killed after 24 h of incubation at T = 37 °C corresponds to the MBC. The estimation of the MBC/MIC ratio describes the bactericidal effect (MBC/MIC < 4) or bacteriostatic (MBC/MIC ≥ 4) of the studied EO.

### 3.6. Determination of the Insecticidal Activity

#### 3.6.1. Animal Matter

The insecticidal property of the EO of *O. elongatum* was studied against adults of *Ceratitis capitata*.

#### 3.6.2. Preparation of the Concentrations

Pure essential oils are dissolved in dimethylsuloxide to create the doses for each plant. From this initial concentration (100 µL/mL), a variety of concentrations of 50, 25, 12.5, 6.25, 3.125, and 1.562 µL/mL were created. Dimethyl sulfoxide is the single component of the control. The bioassay is conducted in a lab setting with a temperature of 28 °C. A total of 10 fruit fly adults are injected into each 400 mL box using a pump after circular sponges with a diameter of 5 cm and a thickness of 0.5 cm are placed in the various concentrations. The coverings of the boxes are hermetically sealed with mosquito nets that have been fastened to create a circle in the middle for better observation control and ventilation assurance. A control with the same number of repetitions was performed to examine the impact of the dimethylsulfoxide. For each essential oil, the bioassay was conducted three times under identical circumstances. Therefore, there are a total of 12 repetitions for each concentration.

### 3.7. Statistical Analysis

The evaluation of the antioxidant activity of the tested EO using the three methods as well as the antibacterial activity (diameter of inhibition) was performed with an analysis of the variance (ANOVA). GraphPad Prism 8 for Windows was used to determine the means and standard deviations. With the Tukey post hoc test, analysis of the variance (ANOVA) was carried out to determine the significance. To compare the mean, a probability threshold of 5% was employed.

## 4. Conclusions

The current investigation showedthat the leaves of *O. elongatum* collected from Bouiblane mountain, Middle Atlas (Morocco) are an important source of EO. Its yield is 4.46%, significantly higher than those reported previously. The chromatographic analysis revealed that carvacrol (57.32%), was the major component of the oil. The essential oil exhibited promising antioxidant, antibacterial and insecticidal activities.The EO’s considerable antioxidant, antibacterial, and insecticidal actions may be attributed to a synergy between the major compound “carvacrol” and the EO’s other minority constituents. The essential oil of *O. elonagtum* has the potential to be a novel alternative source of natural antioxidant, antibacterial, and insecticidal compounds.

## Figures and Tables

**Table 1:** Chemical composition of the EO of *O. elongatum* from Morocco.

N°	Compounds	IK	*O. elongatum*
1	*α*-Thujene	930	0.23
2	*α*-Pinene	939	0.75
3	Camphene	954	0.12
4	Octen-3-ol	979	0.61
5	β-Pinene	979	0.09
6	Myrcene	990	1.43
7	*α*-Phellandrene	1009	0.19
8	*α*-Terpinene	1017	1.10
9	*p*-Cymene	1024	14.70
10	Limonene	1029	0.37
11	1.8-Cineole	1031	0.31
12	*γ* -Terpinene	1059	9.84
13	cis-4-thujanol	1070	0.04
14	Terpinolène	1088	0.12
15	*p*-cymenene	1091	0.12
16	Linalool	1096	2.94
17	Terpinen-4-ol	1177	0.28
18	*α*-Terpineol	1188	0.4
19	Thymol,methyl ether	1235	0.18
20	Thymol	1290	2.71
21	Carvacrol	1299	57.32
22	Caryophyllene *<*(*Z*)>	1412	2.38
23	α-Humulene	1500	23
24	α –Muurolene	1500	0.18
25	*γ*-Cadinene	1523	0.22
26	Thymohydroquinone	1555	0.29
27	Caryophylleneoxide	1583	2.01
**Monoterpene hydrocarbons**			29.06
**Oxygenated monoterpenes**			65.08
**Sesquiterpene hydrocarbons**			2.93
**Oxygenated sesquiterpenes**			2.01
**Total identified compounds**			99.08

**Table 2:** Antioxidant activity of the EO of *O. elongatum* and BHA (Butylated_hydroxyanisole) measured by DPPH, FRAP and TCA methods.

Methods	*O. elongatum* (µL/mL)	BHA (mg/mL)
CI_50_DPPH	2.855 ± 0.018 ****	1.512 ± 0.005 ****
EC_0.5_ FRAP	0.124 ± 0.013 *	0.15 ± 0.002 *
TAC (mg EAA/g HE)	14,099 ± 0.389 *	15.041 ± 0.05 *

The results are significantly different (* *p* < 0.05 and **** *p* < 0.0001).

**Table 3:** Antibacterial activity of the EO of *O. elongatum* and antibiotics (FOX30,TIM85, PRL100) (agar diffusion method) expressed in terms of inhibition zone (mm) [mean ± standard deviation].

Strains	*O. elongatum*	FOX30	TIM85	PRL100
*Enterococcus faecalis*	20.85 ± 1.06	0 ± 0.00	0 ± 0.00	9 ± 0.00
*Serratia fonticola*	45.35 ± 0.919	0 ± 0.00	0 ± 0.00	0 ± 0.00
*Staphylococcus aureus*	9.00 ± 0.00	0 ± 0,00	0 ± 0.00	0 ± 0.00
*Acinétobacter baumannii*	20.70 ± 1.67	21 ± 0,00	15 ± 0.00	10.5 ± 0.00
*Klebsiella oxytoca*	48.05 ± 1.34	0 ± 0,00	0 ± 0.00	11 ± 0.00
*Klebsiella pneumoniae sensible*	14.15 ± 0.212	12 ± 0.00	0 ± 0.00	0 ± 0.00
*E.coli sensible*	45.60 ± 0.566	22 ± 0,00	9 ± 0,00	0 ± 0.00
*E.coli resistante*	14.65 ± 0.495	0 ± 0.00	0 ± 0.00	0 ± 0.00
*Enterobacter aerogenes*	28.90 ± 0.424	20 ± 0.00	14 ± 0.00	10 ± 0.00

The experience was performedin at least two replicates; all results are significantly different from each other (*p* < 0.0001).

**Table 4:** Minimal inhibitory concentration (MIC), minimal bactericidal concentration (MBC), MBC/MIC report values (µL/mL) of *O. elongatum*.

Bacterial Strains			
	MIC	MBC	MBC/MIC
*E. aerogenes*	4	8	2
*Staphylococcus aureus*	32	32	1
*E. faecalis*	4	8	2
*E. coli* sensible	4	4	1
*E. coli*résistante	16	32	2
*K. oxytoca*	8	16	2
*S. fonticola*	4	8	2
*K. pneumoniae* sensible	2	4	2
*A. baumannii*	4	4	1

**Table 5:** DL_50_ of the EO of *O. elongatum* against the adults of *C. capitata*.

EO	Duration of Exposure	
*O. elongatum*	24 h	48 h
	17 ± 0.53	10 ± 0.23

## Data Availability

Data are available upon request.
